# How weak twining lianas adapt to competition with host tree trunks: Case of *Merremia boisiana*

**DOI:** 10.1002/ece3.8800

**Published:** 2022-04-01

**Authors:** Liang Hu, Yuwei Lin

**Affiliations:** ^1^ Geography and Planning School Sun Yat‐sen University Guangzhou China

**Keywords:** anomalous secondary growth, competition, twining liana, vessel density, vessel diameter

## Abstract

Fierce competition exists between most stem‐twining lianas and the trunks of host trees. However, *Merremia boisiana*, a vigorous invasive twining liana, never strangles the host tree. Here, we investigated how *M. boisiana* stems adjust their twining growth to avoid intense competition with host trees, and how hydraulic conductivity is maintained for rapid asexual reproduction. We evaluated the effects of competition on twining *M. boisiana* stems (E_m_) and host tree trunks (E_h_), compared differences in secondary growth between twining and creeping *M. boisiana* stems, calculated the total number of vessels (N_t_), vessel density (Vmm^−2^), average vessel diameter (VD_ave_), and percentage of vessels wider than 300 μm in diameter (P_300_) in the secondary xylem, and traced how these parameters change with increasing cross‐sectional area of stem (SA). The results showed that twining *M. boisiana* stems were competitively weaker, and mean E_m_ (14.3%) was 21 times greater than that of E_h_ (0.7%). Secondary growth along the normal direction of the contact surface was significantly inhibited in stems twining on host trees. The lateral secondary growth of these stems was active, forming secondary vascular rings and/or arcs with abundant large vessels. Secondary growth in the central vascular cylinder was also significantly limited in extremely flat twining stems. N_t_ was positively and linearly correlated with SA. Vmm^−2^ and VD_ave_ fluctuated greatly in younger stems and tended to be stable in older stems. N_t_ and Vmm^−2^ did not significantly differ between twining and creeping stems, while VD_ave_ and P_300_ were both higher in twining stems compared to creeping stems of the same size. In conclusion, well‐developed lateral anomalous secondary growth prevents twining *M. boisiana* stems from fiercely competing with their host trees, while stable vessel density and wider, newly formed, vessels ensured sufficient hydraulic conductivity for the rapid asexual reproduction of twining *M. boisiana* stems.

## Graphical abstract

The total number of vessels in secondary xylem (N_t_) was positively and linearly correlated with cross‐sectional area of stem. Vessel density (Vmm^‐2^), average vessel diameter (VD_ave_), and percentage of vessels wider than 300 μm in diameter (P_300_) fluctuated greatly in younger stems and tended to be stable in older stems.

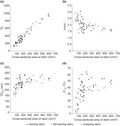

## 1 INTRODUCTION

Lianas (woody climbers) are a principal ecological component of tropical and subtropical forests (Tang et al., 2012). They are structural parasitic plants that achieve upward growth by relying on the trunks and branches of self‐supporting host plants (Stevens, 1987). The interactions between lianas and host trees are of great interest to ecologists (Putz, 1980; Schnitzer & Bongers, 2002). The impacts of tendril‐, hook‐, root‐, and scandent lianas on the trunks of host trees are usually not serious; however, fierce competition exists between most stem‐twining lianas and the trunks of host trees (Qu, 1964; Stevens, 1987). Qu (1964) suggested that competition between liana stems and host tree trunks has three possible scenarios: (1) the twining liana outcompetes the host tree by strangling it (strong liana vs. weak host); (2) the host tree wins and strangles the pest liana by galls, burls, and other abnormal swellings (weak liana vs. strong host); and (3) the two in‐contact species are well‐matched in strength and coexist hard (strong liana vs. strong host).

*Merremia boisiana* (Gagnep.) Oostr. is a perennial convolvulus liana native to west Indonesia, Malaysia, Vietnam, Laos, and south China (Staples, 2010; Wang et al., 2005). In the past three decades, this vigorous invasive species has caused serious damage to the forests and ecosystems of Hainan and Guangdong in China (Li & Huang, 1996; Wang et al., 2009). Climbing capacity enables *M. boisiana* stems ascend up to a height to compete for light and niche with host trees. It mainly infests forest edges and gaps, ascending on suitable host branches or tree trunks, forming a heavy cover on the tree canopy and causing the death of many host trees (Wang et al., 2009). The competition between *M. boisiana* and host trees at the forest edges may involve many biotic and abiotic environmental factors, and the reasons for the death of host trees may also be multi‐faceted, but the situation in the depths of closed forests may be relatively simple. Wang et al. (2009) reported that the thick twining *M. boisiana* stems may also strangle the host tree. However, our observations in South China show that *M. boisiana* coexist well with host trees in the depths of closed forests and the host tree trunks grow normally with no abnormal swelling induced by the twining of *M. boisiana* stems. This interspecies relationship between *M. boisiana* and its host trees did not end with any of the three scenarios of Qu (1964). In this scenario, twining *M. boisiana* stems appeared to be competitively weaker, but had special strategies to avoid intense competition with host trees.

The success of *M. boisiana* is mainly attributed to its rapid rate of asexual reproduction (Cheng et al., 2012; Fan et al., 2016; Li et al., 2006). The rate of asexual reproduction in lianas is restricted by the hydraulic conductivity of the xylem, which is primarily influenced by the size and number of conductive vessels (Ewers et al., 1991; Olson et al., 2014). The maximum and average diameter of vessels in lianas tends to be greater than that of closely related species of self‐supporting plants (Carlquist, 1991; Ewers et al., 1990; Ewers et al., 1997; Fisher & Ewers, 1995; Rosell & Olson, 2014). For example, the VD_ave_ of convolvulus lianas (256 μm) is about five times greater than that of shrubs (50 μm) (Carlquist & Hanson, 1991). Furthermore, the vessels of tropical lianas may remain conductive for many decades (Ewers et al., 1991). Still, lianas must grow new secondary xylem to enhance hydraulic conductivity (Hu & Zhang, 1993; Le et al., 2012; Yang et al., 2014). It is reasonable to speculate that the secondary growth of weak twining *M. boisiana* stems was greatly changed. However, the extent of these changes and the ecological and physiological significance of these changes are still unclear.

In the present study, we aimed to clarify how the secondary structure of *M. boisiana* stems adapt to twining growth to avoid intense competition with host trees, and how twining stems ensure sufficient hydraulic conductivity for rapid asexual reproduction. Specifically, we measured the effects of interspecific competition on the twining stems of *M. boisiana* and the trunks of host trees, compared the stem cross sections and vessel characteristics of twining and creeping *M. boisiana* stems, and traced how vessel characteristics changed as the stem aged. Our results are expected to provide new insight on ecological competition between lianas and their host plants.

## 2 MATERIALS AND METHODS

**FIGURE 1:**
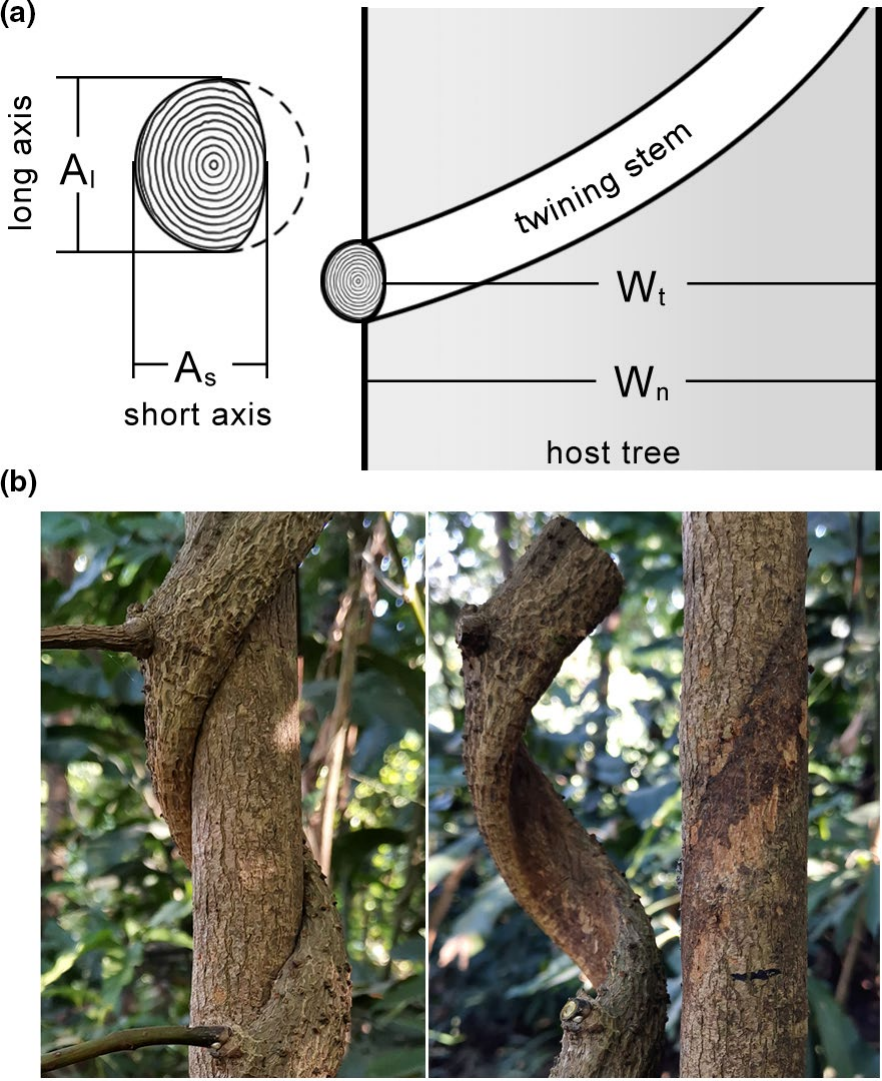
Illustrations of the parameters measured to calculate the effects of interspecific competition between lianas and host trees. (a) A_l_ and A_s_ denote the lengths of the long and short axes in stem cross sections of *Merremia boisiana*, respectively; W_t_ denotes the width of the host tree trunk after being entwined by liana stems; and W_n_ denotes the width of the host tree trunk immediately below the contact point. (b) A case of an extremely flattened twining *M. boisiana* stem before and after removal from the host tree. The twining stem leaves only a very shallow uniform mark on the host tree trunk

Samples of *M. boisiana* were collected from the Longdong Forest Park (N 23°12′38″, E 113°35′49″) in Guangzhou, Pearl River Delta, China. We measured the parameters of twining *M. boisiana* stems and their host tree trunks in the field, following the procedures shown in Figure 1a. The effects of interspecific competition on twining *M. boisiana* stems (E_m_) and host tree trunks (E_h_) were calculated as E_m_ = (A_l_ − A_s_) / A_l_, and E_h_ = (W_n_ − W_t_) / W_n_, where A_l_ and A_s_ denote the lengths of the long and short axes of stem cross sections, respectively; W_t_ denotes width of the host tree trunk after being twined by liana stems; and W_n_ denotes the width of the host tree trunk immediately below the contact point. Host trees infected with a single twining *M. boisiana* stem are difficult to come across, as most host trees suffered from many twining *M. boisiana* stems at the same time, making the sample we can actually measure very limited. In addition, we also had to exclude some samples such as young twining stems born in the current year and host trees badly infected by *M. boisiana* in the canopy. Finally, we measured only 16 valid samples within the study area. As we noted above, the host tree trunks infected with *M. boisiana* stems did not show any abnormal swelling, which means that the activity of the cambium in the host tree trunks was not significantly affected. Besides, the effect of interspecific competition on host tree trunks is relatively uniform (Figure 1b). Therefore, we only measured each selected sample once.

Initial examinations showed that the narrowest vessels in *M. boisiana* stems were mainly limited to the primary xylem, with most being <20 μm diameter. These narrow vessels contribute very little to total hydraulic conductivity according to Poiseuille's law (Chiu & Ewers, 1992; Jiménez‐Castillo & Lusk, 2013; Quintana‐Pulido et al., 2018). The flow rate of an ideal capillary is proportional to the fourth power of capillary diameter; thus, if a capillary doubles in diameter, its hydraulic transport rate could theoretically increase 16‐fold (Ewers et al., 1989, 1991; Zimmermann, 1983). The theoretical rate of flow of a vessel that is 100 μm in diameter is equivalent to the sum of the rate of flow in 10,000 vessels of 10 μm diameter. Therefore, hydraulic conductivity in liana stems is mainly regulated by wide vessels in the secondary xylem, with the many narrow vessels carrying insignificant amounts of water and contributing very little to total hydraulic conductivity (Ewers et al., 1989; Zimmermann, 1983). For example, the largest 6.5% of vessels in *Cissus striata* were responsible for almost 50% of total theoretical hydraulic conductivity (Jiménez‐Castillo & Lusk, 2013). In contrast, vessels smaller than 18 μm contributed less than 1% of theoretical hydraulic conductivity in *Lonicera japonica* (Chiu & Ewers, 1992). Vessels smaller than 60 μm only contributed to 3–6% of total theoretical hydraulic conductivity in *Vitis vinifera* (Quintana‐Pulido et al., 2018). Therefore, only vessels in the secondary xylem were counted and evaluated in this study.

We collected samples of undamaged twining and creeping stems of various diameters. Samples of two types of twining stems was collected: (1) stems that twined on the host tree and (2) stems that entwined together. For each sample, we selected intact slices of stem cross sections. We placed each slice on a glass slide with microscope reticle, placed a concave mirror below the glass slide to allow natural light to pass through the slice from below, and took pictures with a macro camera from above along the normal direction. The vessels were clearly shown as white circles in the photographs. Photographs were then processed in Photoshop 8.0 CS (Adobe Inc., 2003, San Jose, USA). The boundaries of vessels and stem cross sections were extracted and converted to a vector graph for output. The cross‐sectional area of stem (SA) and area of each vessel (VA) were then calculated in AutoCAD 2010 (Autodesk, 2009, San Rafael, USA). The vessel diameter (VD) of each vessel was calculated using the equivalent circle area diameter. The total number of vessels in stem cross section (N_t_) and the number of vessels in the central vascular cylinder (N_c_) were counted, respectively. The average vessel diameter (VD_ave_), vessel density (Vmm^−2^, number of vessels per mm^2^), and percentage of vessels wider than 300 μm in diameter (P_300_) were calculated for each stem. All data analyses were calculated in Microsoft Excel 2019 for Windows (Microsoft, 2018, Washington, USA), and final vector graphics were drawn in Adobe Illustrator CC 2017 (Adobe Inc., 2016, San Jose, USA).

## 3 RESULTS

### 3.1 Effects of interspecific competition

Twining *M. boisiana* stems usually left only a very shallow mark on host tree trunks. The average effect of interspecific competition on host tree trunks (E_h_) was just 0.7% (*n* = 16) (Table 1). In contrast, the effect of competition on twining *M. boisiana* stems (E_m_) was much higher compared to E_h_ in most cases. Overall, the average E_m_ (14.3%) was 21.0 times greater than that of E_h_, with a maximum of 56.2% (Table 1). The average E_m_ of the eight thinner twining stems (<1 cm in diameter) was only 2.9%, significantly lower (*p* < .01) than that of the eight thicker stems (25.6%).

**TABLE 1:** Effects of interspecific competition on twining *Merremia boisiana* stems (E_m_) and host tree trunks (E_h_)

No.	Twining stem (mm)		Host tree (mm)		Effect (%)		Host species
	A_l_	A_s_	W_n_	W_t_	E_m_	E_h_	
1	3.4	3.3	26.2	26.2	2.9	–	*Aquilaria sinensis*
2	3.4	3.2	31.1	30.5	5.9	1.8	*A. sinensis*
3	3.7	3.7	7.7	7.7	–	–	*Eucalyptus robusta*
4	3.9	3.8	12.4	12.1	2.6	1.9	*E. robusta*
5	5.3	5.0	22.8	22.4	5.7	1.4	*E. robusta*
6	6.2	6.0	67.9	67.6	3.2	0.4	*Schima superba*
7	6.5	6.3	95.9	95.9	3.1	–	*Tetradium glabrifolium*
8	7.2	7.2	80.4	79.6	–	0.9	*Toxicodendron succedaneum*
9	11.5	10.5	45.0	44.3	8.1	0.1	*A. sinensis*
10	13.3	12.1	94.1	94.0	8.6	0.7	*A. sinensis*
11	18.4	15.9	175.7	173.6	13.6	1.1	*Canarium album*
12	21.1	13.4	184	182.9	36.5	0.6	*S. superba*
13	25.5	25.3	83.8	82.4	0.8	1.3	*E. robusta*
14	26.5	11.6	30.6	30.3	56.2	0.7	*S. superba*
15	30.0	21.4	16.2	16.2	28.7	–	*Vitex quinata*
16	32.4	15.4	182.2	182	52.5	0.1	*S. superba*

Note

A_l_ and A_s_ denote long and short axes in cross section of the twining *M. boisiana* stem. W_n_, normal width of host tree; W_t_, width of host tree twined by *M. boisiana*; E_m_ = (A_l_ − A_s_) / A_l_; E_h_ = (W_n_ − W_t_) / W_n_.

### 3.2 Anomalous secondary growth

Eighty‐one cross sections of central vascular cylinder of *M. boisiana* stems were analyzed, including 29 creeping stems, 37 single twining stems with secondary vascular rings, four extremely flat twining stems with secondary vascular arcs, and 11 entwined twining stems.

**FIGURE 2:**
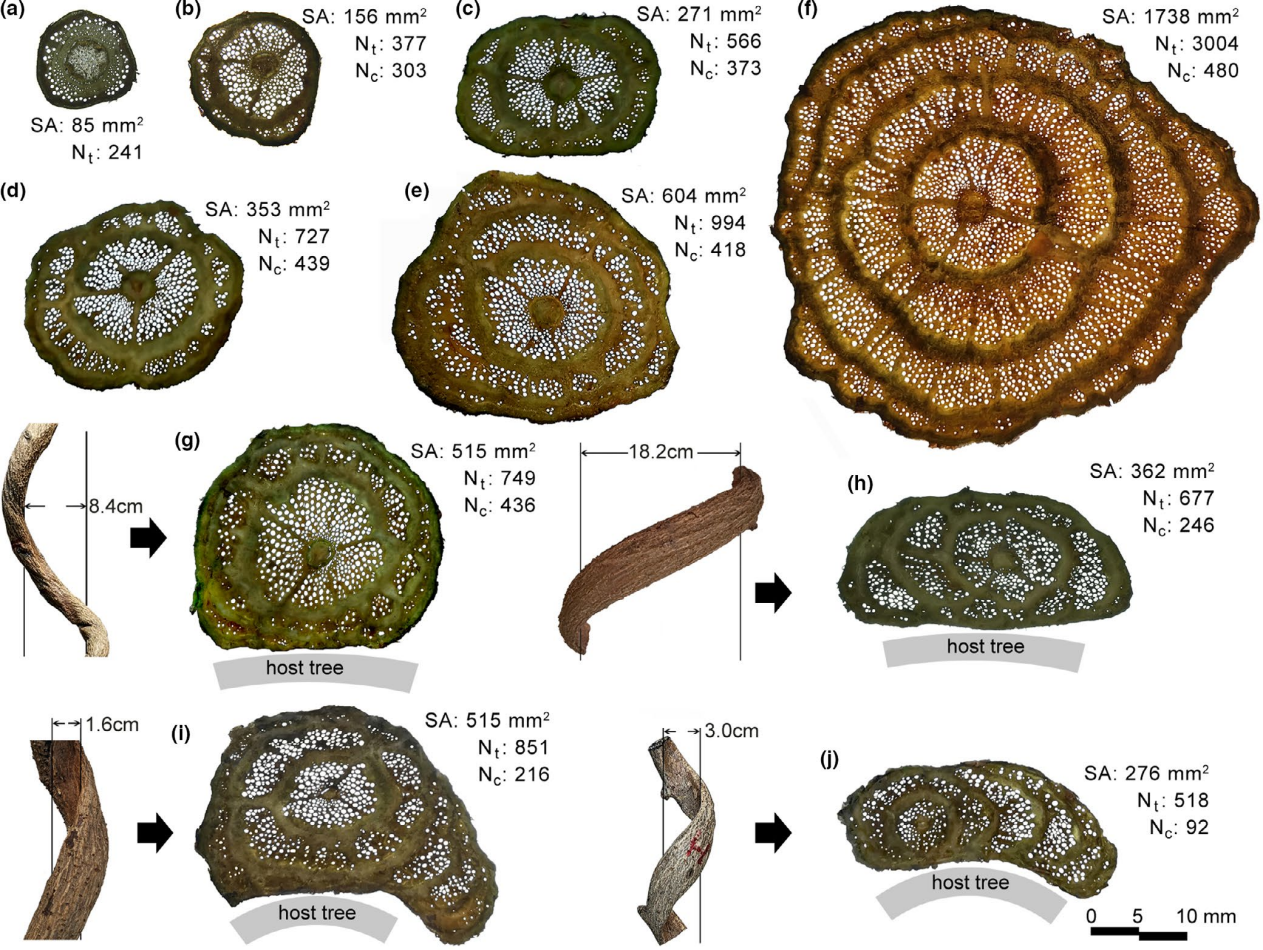
Cross sections of *Merremia boisiana* stems showing anomalous secondary vascular rings and arcs. Material in (a) to (f) was obtained from different parts of a single creeping stem. Material in (g) to (j) was obtained from four different stems twining on host trees

Outermost secondary growth activity in creeping *M. boisiana* stems was not synchronous in all directions, with some directions growing earlier than others; however, concentric secondary vascular rings were eventually formed that were separated by conjunctive tissues (Figure 2a–f). In comparison, secondary growth in twining *M. boisiana* stems along the normal direction of the contact surface was more or less limited (Figure 2g–j). In some cases, lateral secondary growth tended to be more active on one side, generated many lateral secondary vascular arcs, and formed extremely flat twining stems (Figure 2h–j).

**FIGURE 3:**
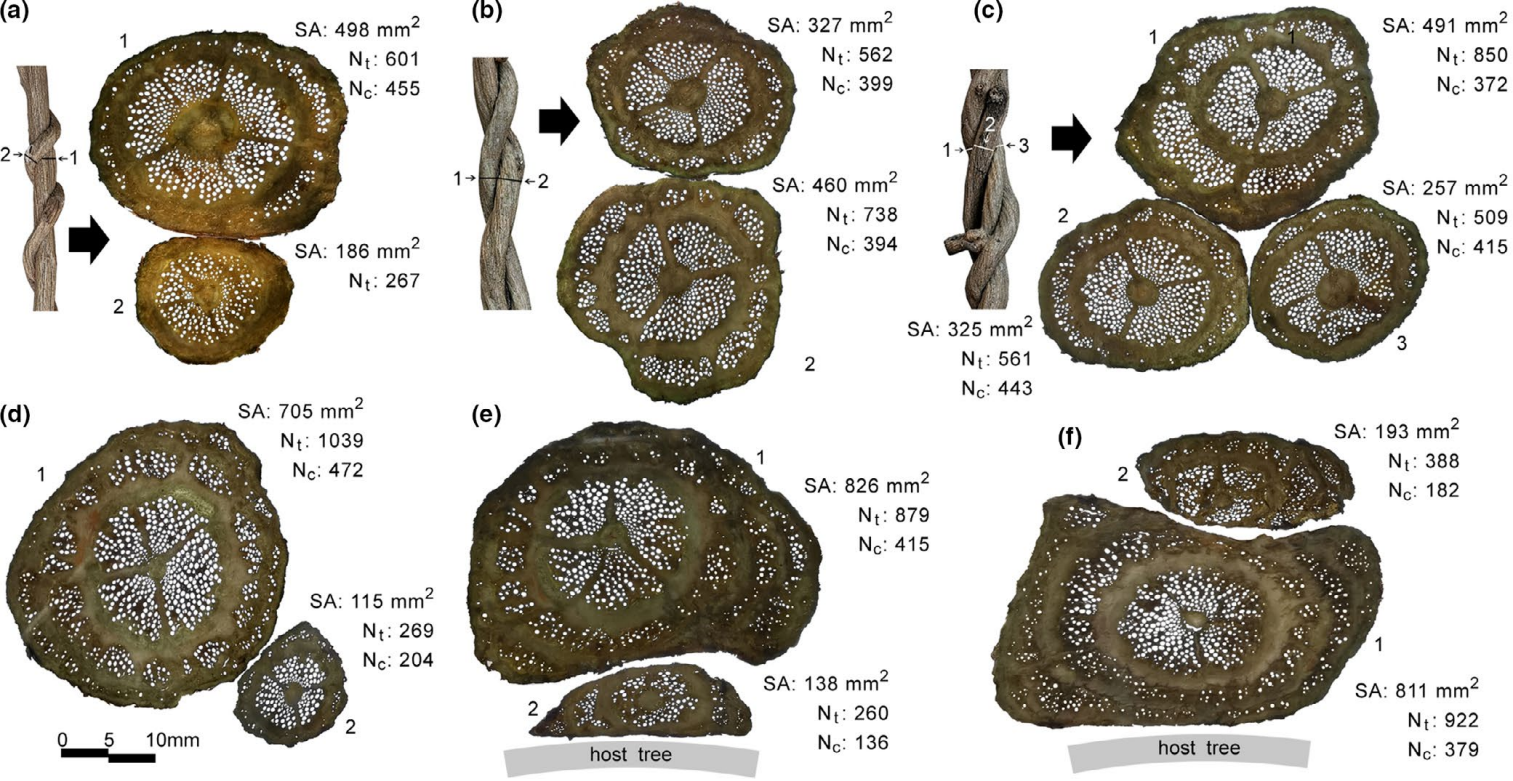
Cross sections of entwined *Merremia boisiana* stems showing anomalous vascular rings and arcs. (a) Thinner stem twined on a thicker stem; (b) two stems entangled with each other; (c) three entangled stems; (d) two entwined stems not yet entangled in host tree; (e) two entwined stems entangled in host tree, with the thinner one inside; (f) two entwined stems entangled in host tree, with the thicker one inside. Material in (d) to (f) was obtained from different parts of two entwined stems. The stems entwined each other and then entangled in a host tree together

Anomalous secondary growth of entwined *M. boisiana* stems was similar to that of creeping stems and was not significantly inhibited by each other (Figure 3a–d). In contrast, when two *M. boisiana* stems entwined each other and then twined with the same host tree, secondary growth was extremely limited both in the central vascular cylinder and along the normal direction of the contact surface. This situation was exacerbated if one of them twisted in between the host tree trunk and the other twining stem (Figure 3e,f). Of note, we only identified one and two abnormal vascular rings, respectively, in the two entwined stems before they entangled with the host tree (Figure 3d); however, there were two and three rings/arcs, respectively, in their deformed stems that entangled with the host tree trunks (Figure 3e,f).

**FIGURE 4:**
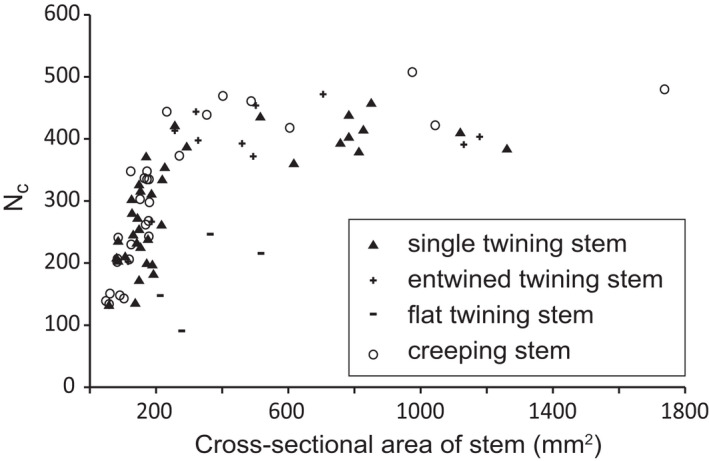
Number of vessels in the secondary xylem of the central vascular cylinder (N_c_) of twining and creeping *Merremia boisiana* stems

The number of vessels in the central vascular cylinder (N_c_) increased as the stem aged and tended to stabilize in mature stems (Figure 4). No significant differences were observed between single twining, entwined twining, and creeping groups in N_c_. However, N_c_ in extremely flat twining stems was significantly lower than that in other stems of the same size (Figure 4).

### 3.3 Density and diameter of vessels

**FIGURE 5:**
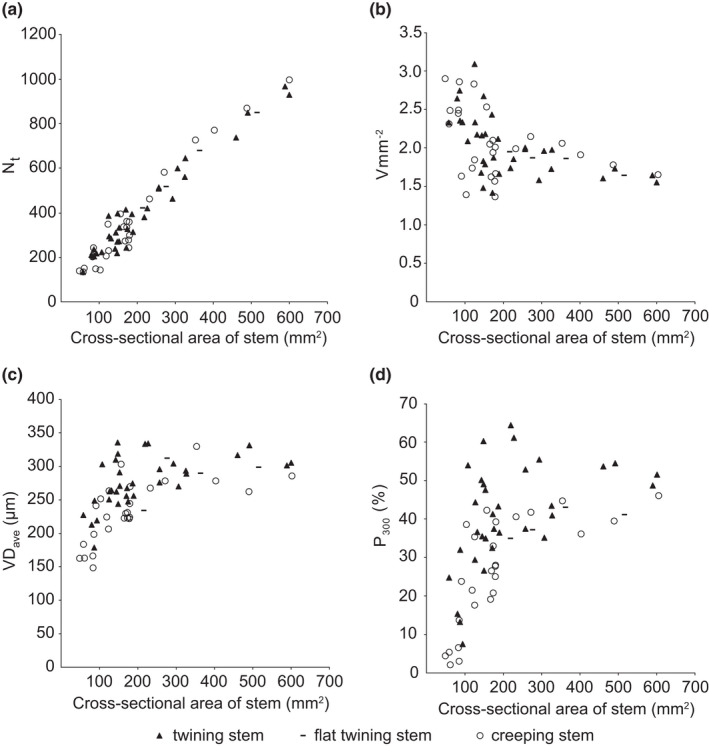
Characteristics of vessels in the secondary xylem of twining and creeping *Merremia boisiana* stems. Extremely flat twining stems were separately grouped. (a) Total number of vessels (N_t_); (b) number of vessels per mm^2^ (Vmm^−2^); (c) average vessel diameter (VD_ave_); and (d) percentage of vessels wider than 300 μm in diameter (P_300_)

Sixty‐three cross sections of entire *M. boisiana* stems were analyzed, including 37 twining and 26 creeping stems. No significant differences were observed between extremely flat twining stems and the rest twining stems (Figure 5a–d). Overall, the total number of vessels in secondary xylem (N_t_) was positively and linearly correlated with cross‐sectional area of stem (SA) (Figure 5a). Vessel density (Vmm^−2^) fluctuated greatly in younger stems (lower than 200 mm^2^ in SA) and tended to be stable in older stems (Figure 5b). No significant differences were observed between twining and creeping groups in N_t_ and Vmm^−2^. Similarly, average vessel diameter (VD_ave_) and percentage of vessels wider than 300 μm in diameter (P_300_) exhibited greater variance in younger stems and tended to be stable in older stems (Figure 5c–d). However, VD_ave_ and P_300_ was higher in twining stems compared to creeping stems of the same SA (Figure 5c–d). For example, VD_ave_ was higher than 300 μm in 12 (32.4%) twining stems and P_300_ was higher than 50% in ten (27.0%) twining stems, while VD_ave_ was higher than 300 μm in only two (7.7%) creeping stems and P_300_ was higher than 50% in only one (3.8%) creeping stem. The maximum values of VD_ave_ (335 μm) and P_300_ (64%) were both recorded in twining stems.

**FIGURE 6:**
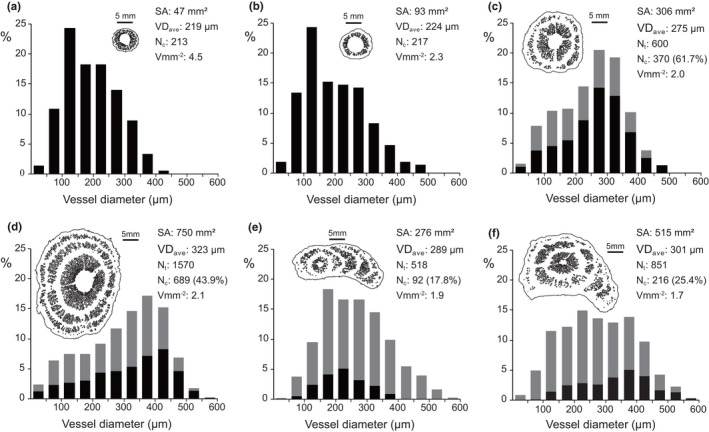
Frequency distribution of vessel diameter in *Merremia boisiana* stems. The first four cross sections were obtained from different parts of a single twining stem, and the last two cross sections were obtained from two other different twining stems. (a) Young stem without conjunctive tissue; (b) young stem with conjunctive tissue; (c) stem with one anomalous vascular ring; (d) stem with two anomalous vascular rings; (e) stem with many lateral anomalous vascular arcs; (f) stem with one anomalous vascular ring and many lateral anomalous vascular arcs. Black column denotes vessels in the central vascular cylinder, gray column denotes vessels in the anomalous vascular rings and/or arcs. SA: cross‐sectional area of stem; VD_ave_: average vessel diameter, N_c_: number of vessels in the central vascular cylinder; N_t_: total number of vessels in the stem cross section. Vmm^−2^: vessel density (number of vessels per mm^2^)

We examined changes to the frequency distribution of vessel diameter over four stages of a single *M. boisiana* stem twining on a large tree (Figure 6a–d). In the central vascular cylinder, the number of vessels (N_c_) and frequency of large vessels increased with increasing SA (Figure 6a–d). A similar pattern was detected for anomalous secondary vascular rings. As a result, N_t_, VD_ave_, and the frequency of large vessels increased with increasing age; thus, the mean diameter of the newly formed vessels was wider than that of already formed ones. The P_300_ increased from 12.7% to 16.1%, 34.7%, and 55.7% over the four stages of growth. Vmm^−2^ was greater in younger stems compared to older stems, due to the presence of less conjunctive tissues (Figure 6a).

For extremely flat twining stems, anomalous secondary growth in their central vascular cylinder was clearly suppressed, and the ratio of N_c_ over N_t_ was significantly lower than that of stems twining on large trees (Figure 6e,f). However, the Vmm^−2^ of these extremely deformed stems tended to be stable, due to the abundance of large vessels in the anomalous secondary vascular rings and/or arcs (Figure 6e,f). The P_300_ was 35.3% in the smaller stem and 42.7% in the larger stem.

## 4 DISCUSSION

In most cases, competition between lianas and their hosts determines survival (Qu, 1964); however, an alternative tactic was displayed by *M. boisiana*. The host trees being entangled were able to grow normally, with the liana having a negligible impact on the radial growth of the tree trunk, indicating that the death of the host trees was not due to the strangulation of the twining *M. boisiana* stems. The E_h_ detected in this study was probably limited to bark and had no actual effect on the vascular cambium of host trees. Twining *M. boisiana* stems were confirmed to be competitively weaker; however, they had two strategies to overcome those disadvantages: active lateral anomalous secondary growth and wide vessels.

### 4.1 Anomalous secondary growth

Well‐developed lateral anomalous secondary growth was a successful strategy to help weak twining *M. boisiana* stems ensured sufficient hydraulic conductivity and avoided fiercely competing with the trunks of host trees. Anomalous secondary growth may provide both hydraulic and mechanical advantages for lianas (Isnard & Field, 2015). Anomalous secondary growth along the normal direction of the contact surface was limited in twining *M. boisiana* stems. Instead, lateral secondary growth dominated and formed secondary vascular arcs. Similar phenomena were also reported in the stems of other liana species (Cabellé, 1993; Fisher & Ewers, 1991; Isnard & Field, 2015). Our results showed that the secondary growth in the central vascular cylinder of twining *M. boisiana* stem might be limited, especially in extremely flat twining stems; however, N_t_, VD_ave_, Vmm^−2^, and P_300_ of the stems remained unaffected. It is not clear what caused the flattening of these lianas. One advantage of lateral secondary vascular arcs may be that the newly formed vessels do not have to coordinate with extremely twisted stems and avoid being squeezed by the already formed liana stem and the trunk of host trees.

Abnormal secondary vascular rings in liana stems differ the growth rings produced by the periodic growth of trees and shrubs (Hu & Zhang, 1993; Lima et al., 2010; Zhang & Hu, 1987). For example, *Phytolacca acinosa* roots produced seven abnormal vascular rings in three years (Zhang & Hu, 1987). According to Carlquist and Hanson (1991), the cambia of Convolvulaceae species do not form annually. Ye et al. (2006) treated the anomalous secondary vascular rings of creeping *M. boisiana* stems as growth rings to record annual growth rates. Our results showed that the number of lateral secondary vascular arcs did not match with the number of secondary vascular rings in intertwined *M. boisiana* stems, indicating that rings and arcs of twining *M. boisiana* stems did not form annually. *M. boisiana* could grow year‐round in south China, with new shoots growing more than 16 m per year in length, peaking in April and September (Fan et al., 2016). However, there is no evidence that radial activity of successive cambia in *M. boisiana* stems is synchronized with elongation growth. It remains unclear whether the secondary vascular rings in creeping *M. boisiana* stems are related to periodic growth. The anomalous secondary growth rhythm of *M. boisiana* stems requires further study.

### 4.2 Wide vessels

Twining *M. boisiana* stems have outstanding advantages in vessel size compared with many other twining lianas. In secondary xylem of mature twining *M. boisiana* stems, the VD_ave_ was higher than the average VD_ave_ of convolvulus lianas reported by Carlquist and Hanson (1991), and the P_300_ was higher than that of all native lianas in the Pearl River Delta reported by Hu (2009). Vessels in the primary xylem were not evaluated in the present study; consequently, the Vmm^−2^ in the secondary xylem of mature *M. boisiana* stems was lower than that of mature convolvulus lianas (3–15) reported by Carlquist and Hanson (1991) and herbaceous convolvulus vines (3.5–13.2) reported by Rosell and Olson (2014). However, conclusions on hydraulic conductivity could not be drawn without the frequency distribution of vessels. Of note, the VD_ave_, Vmm^−2^ and P_300_ of *M. boisiana* were stable in older stems, but fluctuated greatly in younger stems. Unfortunately, these differences between younger and older stems had not been taken seriously in previous studies (e.gCarlquist & Hanson, 1991; Hu, 2009; Rosell & Olson, 2014). Their conclusions of interspecific comparison based on samples with significantly varying stem sizes were doubtful.

Moreover, vessel elements are not ideal capillaries, with flow rate being limited by vessel resistivity (Zimmermann, 1983). Because vessel resistivity decreases strongly with increasing vessel diameter (Christman & Sperry, 2010), wider vessels might enhance actual flow rate more than estimated. Actual flow rate also depends on other factors, including gravity, solute content, liquid viscosity, and root pressure (Ewers et al., 1991; Zimmermann, 1983). Consequently, measured flow rates tend to be significantly lower than the theoretical values predicted by Poiseuille's law (Chiu & Ewers, 1992; Ewers et al., 1989; Gloser et al., 2011). However, it is unclear whether these factors aggravate different flow rates between wide and narrow vessels. In conclusion, considering the huge differences in theoretical flow rate between wide and narrow vessels, both VD_ave_ and Vmm^−2^ are not ideal parameters for comparing the hydraulic conductivity of different species, even when the narrowest vessels are considered.

Yet, it is appropriate to use VD_ave_ and Vmm^−2^ to track changes to hydraulic conductivity at different growth stages of the same species when the frequency distribution of vessels is also analyzed. The Vmm^−2^ in mature twining *M. boisiana* stems was stable, and the frequency of wide vessels increased as the stem aged; thus, the total hydraulic conductivity of the stem and the average hydraulic conductivity per vessel both increase steadily. The VD_ave_ in twining *M. boisiana* stems is wider than that of creeping stems of the same size; consequently, the total hydraulic conductivity of the stem and hydraulic conductivity per vessel were both higher in the former, when the effects of gravity and root pressure were not considered. Overall, wide vessels are undoubtedly a successful strategy to ensure efficient hydraulic conductivity in twining *M. boisiana* stems.

## 5 CONCLUSIONS

Liana–tree interactions provide important insights on understanding the behavior of lianas and their role in forest ecosystems. Previous studies primarily focused on evaluating the fierce competition between liana stems and host tree trunks. Our results showed the possibility of a weak interaction between fast‐growing lianas and host trees, whereby host trees survive with trunk radial growth not being affected. *Merremia boisiana* stems may respond by having wide vessels and active lateral anomalous secondary growth to adapt twining growth on the trunks of host trees. Our results provided new insights on liana–tree competition. However, this weak interaction between stems and trunks does not represent the final interspecific relationship. Ultimately, competition between lianas and their host trees is not limited to stems and trunks. Our findings should be tested and verified by further studies on other weak stem‐twining lianas, with a strong focus on anomalous secondary growth rhythm of liana stems.

## CONFLICT OF INTEREST

None declared.

## AUTHOR CONTRIBUTIONS

**Liang Hu:** Conceptualization (lead); Data curation (equal); Funding acquisition (lead); Investigation (equal); Project administration (lead); Resources (equal); Software (equal); Supervision (lead); Writing – original draft (lead); Writing – review & editing (equal). **Yuwei Lin:** Data curation (equal); Formal analysis (equal); Investigation (equal); Software (equal); Writing – review & editing (equal).

## Supporting information

Appendix S1

## Data Availability

The data that support the findings of this study are available in the Supplementary Material of this article.
