# Supplementary material for: How weak twining lianas adapt to competition with host tree trunks: Case of Merremia boisiana
Source: Ecol Evol. 2022 Apr 1;12(4):e8800. doi: 10.1002/ece3.8800 (PMC8975785; doi:10.1002/ece3.8800)
Supplement: Supplementary file 1 — Appendix S1 [file ECE3-12-e8800-s001.docx]

**Appendix**

**Table A1** Stem cross-sectional area (SA) and number of vessels in the secondary xylem of the central vascular cylinder (N_c_) of twining and creeping *Merremia* *boisiana* stems.

| **No.** | **Stem type** | **SA/mm^2^** | **N_c_** | **No.** | **Stem type** | **SA/mm^2^** | **N_c_** |
| --- | --- | --- | --- | --- | --- | --- | --- |
| 1 | twining | 57.5 | 134 | 42 | entwined | 498.3 | 455 |
| 2 | twining | 79.5 | 210 | 43 | entwined | 186.0 | 267 |
| 3 | twining | 86.0 | 236 | 44 | entwined | 327.0 | 399 |
| 4 | twining | 86.7 | 204 | 45 | entwined | 460.2 | 394 |
| 5 | twining | 106.8 | 211 | 46 | entwined | 491.3 | 372 |
| 6 | twining | 124.9 | 303 | 47 | entwined | 257.0 | 415 |
| 7 | twining | 126.1 | 281 | 48 | entwined | 705.4 | 472 |
| 8 | twining | 131.1 | 246 | 49 | entwined | 115.1 | 204 |
| 9 | twining | 142.0 | 233 | 50 | entwined | 325.0 | 443 |
| 10 | twining | 143.8 | 273 | 51 | entwined | 1130.3 | 389 |
| 11 | twining | 147.2 | 173 | 52 | entwined | 1176.5 | 401 |
| 12 | twining | 147.6 | 255 | 53 | creeping | 47.9 | 139 |
| 13 | twining | 148.5 | 327 | 54 | creeping | 58.0 | 134 |
| 14 | twining | 152.3 | 226 | 55 | creeping | 60.7 | 151 |
| 15 | twining | 152.7 | 317 | 56 | creeping | 82.5 | 202 |
| 16 | twining | 170.1 | 372 | 57 | creeping | 83.1 | 207 |
| 17 | twining | 171.5 | 200 | 58 | creeping | 85.4 | 241 |
| 18 | twining | 174.5 | 239 | 59 | creeping | 90.6 | 148 |
| 19 | twining | 186.1 | 312 | 60 | creeping | 102.7 | 143 |
| 20 | twining | 188.8 | 198 | 61 | creeping | 118.6 | 206 |
| 21 | twining | 211.2 | 148 | 62 | creeping | 123.3 | 348 |
| 22 | twining(flat) | 216.4 | 262 | 63 | creeping | 124.7 | 230 |
| 23 | twining | 218.6 | 336 | 64 | creeping | 151.6 | 303 |
| 24 | twining | 226.5 | 355 | 65 | creeping | 164.4 | 337 |
| 25 | twining | 256.6 | 422 | 66 | creeping | 168.1 | 262 |
| 26 | twining(flat) | 275.9 | 92 | 67 | creeping | 172.5 | 348 |
| 27 | twining | 292.7 | 389 | 68 | creeping | 172.6 | 335 |
| 28 | twining(flat) | 361.9 | 246 | 69 | creeping | 177.3 | 268 |
| 29 | twining(flat) | 515.0 | 216 | 70 | creeping | 178.1 | 243 |
| 30 | twining | 515.4 | 436 | 71 | creeping | 178.7 | 335 |
| 31 | twining | 615.4 | 361 | 72 | creeping | 179.6 | 298 |
| 32 | twining | 758.0 | 393 | 73 | creeping | 232.3 | 444 |
| 33 | twining | 782.1 | 439 | 74 | creeping | 270.5 | 373 |
| 34 | twining | 784.3 | 406 | 75 | creeping | 353.4 | 439 |
| 35 | twining | 850.0 | 459 | 76 | creeping | 402.0 | 469 |
| 36 | twining | 1121.2 | 412 | 77 | creeping | 487.0 | 461 |
| 37 | twining | 1260.4 | 384 | 78 | creeping | 604.1 | 418 |
| 38 | twining | 826.1 | 415 | 79 | creeping | 973.0 | 507 |
| 39 | twining | 138.0 | 136 | 80 | creeping | 1042.0 | 421 |
| 40 | twining | 193.0 | 182 | 81 | creeping | 1737.6 | 480 |
| 41 | twining | 811.2 | 379 |  |  |  |  |

**Table A2** Characteristics of vessels in the secondary xylem of twining and creeping *Merremia* *boisiana* stems. SA: stem cross-sectional area. SD: Stem diameter. TVA: total vessel area in the stem cross section. NV: total number of vessels in the stem cross section. VD_ave_: average vessel diameter. Vmm^-2^: number of vessels per mm^2^. N_300_: number of vessels wider than 300 μm in diameter.

| **No.** | **Stem type** | **SA/mm^2^** | **SD/mm** | **TVA/mm^2^** | **NV** | **VD_ave_/μm** | **Vmm^-2^** | **N_300_** |
| --- | --- | --- | --- | --- | --- | --- | --- | --- |
| 1 | twining | 57.5 | 8.56 | 5.41 | 134 | 226.7 | 2.33 | 33 |
| 2 | twining | 79.5 | 10.06 | 7.43 | 210 | 212.2 | 2.64 | 32 |
| 3 | twining | 86.0 | 10.46 | 5.89 | 236 | 178.2 | 2.74 | 31 |
| 4 | twining | 86.7 | 10.50 | 9.87 | 204 | 248.3 | 2.35 | 65 |
| 5 | twining | 93.0 | 10.88 | 8.15 | 217 | 218.7 | 2.33 | 16 |
| 6 | twining | 106.8 | 11.66 | 16.02 | 223 | 302.4 | 2.09 | 120 |
| 7 | twining | 124.9 | 12.61 | 18.93 | 386 | 249.9 | 3.09 | 113 |
| 8 | twining | 126.1 | 12.67 | 15.84 | 294 | 261.9 | 2.33 | 130 |
| 9 | twining | 131.1 | 12.92 | 15.54 | 285 | 263.5 | 2.17 | 104 |
| 10 | twining | 142.0 | 13.45 | 17.86 | 238 | 309.1 | 1.68 | 119 |
| 11 | twining | 143.8 | 13.53 | 16.71 | 311 | 261.6 | 2.16 | 110 |
| 12 | twining | 147.2 | 13.69 | 19.19 | 218 | 334.8 | 1.48 | 131 |
| 13 | twining | 147.6 | 13.71 | 21.46 | 270 | 318.1 | 1.83 | 132 |
| 14 | twining | 148.5 | 13.75 | 18.48 | 397 | 243.5 | 2.67 | 105 |
| 15 | twining | 152.3 | 13.92 | 18.02 | 272 | 290.4 | 1.79 | 129 |
| 16 | twining | 152.7 | 13.94 | 19.09 | 333 | 270.2 | 2.18 | 116 |
| 17 | twining | 170.1 | 14.72 | 21.19 | 414 | 255.3 | 2.43 | 134 |
| 18 | twining | 171.5 | 14.78 | 13.59 | 243 | 266.9 | 1.42 | 100 |
| 19 | twining | 174.5 | 14.91 | 15.64 | 327 | 246.8 | 1.87 | 122 |
| 20 | twining | 186.1 | 15.39 | 23.22 | 394 | 273.9 | 2.12 | 170 |
| 21 | twining | 188.8 | 15.51 | 16.08 | 314 | 255.3 | 1.66 | 114 |
| 22 | twining(flat) | 216.4 | 16.60 | 18.31 | 425 | 234.2 | 1.96 | 149 |
| 23 | twining | 218.6 | 16.68 | 33.05 | 380 | 332.8 | 1.74 | 244 |
| 24 | twining | 226.5 | 16.98 | 36.66 | 420 | 333.4 | 1.85 | 256 |
| 25 | twining | 256.6 | 18.08 | 30.30 | 509 | 275.3 | 1.98 | 190 |
| 26 | twining | 256.6 | 18.08 | 35.14 | 514 | 295.0 | 2.00 | 271 |
| 27 | twining(flat) | 275.9 | 18.75 | 39.50 | 518 | 311.6 | 1.88 | 192 |
| 28 | twining | 292.7 | 19.31 | 33.49 | 463 | 303.5 | 1.58 | 256 |
| 29 | twining | 306.0 | 19.74 | 34.20 | 600 | 269.4 | 1.96 | 210 |
| 30 | twining | 325.1 | 20.35 | 37.80 | 561 | 292.9 | 1.73 | 243 |
| 31 | twining | 326.5 | 20.39 | 42.10 | 645 | 288.3 | 1.98 | 263 |
| 32 | twining(flat) | 361.9 | 21.47 | 45.00 | 677 | 290.9 | 1.87 | 292 |
| 33 | twining | 459.9 | 24.20 | 57.90 | 738 | 316.1 | 1.60 | 395 |
| 34 | twining | 490.6 | 24.99 | 73.00 | 850 | 330.7 | 1.73 | 462 |
| 35 | twining(flat) | 515.0 | 25.61 | 59.80 | 851 | 299.1 | 1.65 | 350 |
| 36 | twining | 588.7 | 27.38 | 68.70 | 968 | 300.6 | 1.64 | 470 |
| 37 | twining | 599.8 | 27.64 | 67.80 | 930 | 304.7 | 1.55 | 478 |
| 38 | creeping | 47.9 | 7.81 | 2.87 | 139 | 162.3 | 2.90 | 6 |
| 39 | creeping | 58.0 | 8.59 | 3.53 | 134 | 183.0 | 2.31 | 7 |
| 40 | creeping | 60.7 | 8.79 | 3.13 | 151 | 162.5 | 2.49 | 3 |
| 41 | creeping | 82.5 | 10.25 | 4.36 | 202 | 165.8 | 2.45 | 13 |
| 42 | creeping | 83.1 | 10.28 | 3.57 | 207 | 148.1 | 2.49 | 6 |
| 43 | creeping | 85.0 | 10.40 | 7.48 | 243 | 198.0 | 2.86 | 33 |
| 44 | creeping | 90.6 | 10.74 | 6.74 | 148 | 240.7 | 1.63 | 35 |
| 45 | creeping | 102.7 | 11.44 | 7.06 | 143 | 250.8 | 1.39 | 55 |
| 46 | creeping | 118.6 | 12.29 | 8.10 | 206 | 223.8 | 1.74 | 44 |
| 47 | creeping | 123.3 | 12.53 | 11.63 | 349 | 206.0 | 2.83 | 61 |
| 48 | creeping | 124.7 | 12.60 | 12.48 | 230 | 262.8 | 1.84 | 81 |
| 49 | creeping | 156.0 | 14.09 | 28.39 | 395 | 302.5 | 2.53 | 166 |
| 50 | creeping | 164.4 | 14.47 | 13.06 | 337 | 222.1 | 2.05 | 64 |
| 51 | creeping | 168.1 | 14.63 | 11.25 | 273 | 229.0 | 1.62 | 72 |
| 52 | creeping | 172.5 | 14.82 | 15.11 | 362 | 230.5 | 2.10 | 119 |
| 53 | creeping | 172.6 | 14.83 | 13.01 | 335 | 222.3 | 1.94 | 69 |
| 54 | creeping | 177.3 | 15.03 | 10.70 | 278 | 221.3 | 1.57 | 69 |
| 55 | creeping | 178.1 | 15.06 | 9.54 | 243 | 223.5 | 1.36 | 67 |
| 56 | creeping | 178.7 | 15.08 | 16.77 | 359 | 243.8 | 2.01 | 100 |
| 57 | creeping | 179.6 | 15.12 | 17.00 | 299 | 269.1 | 1.66 | 117 |
| 58 | creeping | 232.3 | 17.20 | 25.85 | 462 | 266.9 | 1.99 | 187 |
| 59 | creeping | 270.5 | 18.56 | 35.23 | 582 | 277.6 | 2.15 | 242 |
| 60 | creeping | 353.4 | 21.21 | 61.78 | 727 | 328.9 | 2.06 | 324 |
| 61 | creeping | 402.0 | 22.62 | 45.00 | 770 | 277.1 | 1.92 | 277 |
| 62 | creeping | 487.0 | 24.90 | 46.20 | 869 | 260.7 | 1.78 | 253 |
| 63 | creeping | 604.1 | 27.73 | 81.00 | 994 | 322.1 | 1.65 | 517 |
